# DaVinci robotic-assisted laparoscopic resection of parapelvic cavernous hemangioma: a case report

**DOI:** 10.1186/s12893-020-00834-4

**Published:** 2020-08-13

**Authors:** Zheng-Jun Chen, Dong Wang, Shi-Da Fan, Shang-Qing Ren, Fang Zhou, Yu Nie, Qian Lv, Jing-Zhi Tian

**Affiliations:** Department of Robotic Minimally Invasive Surgery Center, Sichuan Academy of Medical Sciences & Sichuan Provincial People’s Hospital, Chengdu, 610072 China

**Keywords:** DaVinci surgical system, Cavernous hemangioma, Laparoscopic resection

## Abstract

**Background:**

Cavernous hemangioma, as a rare tumor, is difficult to differentiate from retroperitoneal lymphoma and paraganglioma. They are more difficult to excise completely through open surgery and traditional laparoscopic surgery. The study aimed to evaluate the role of DaVinci surgical system in laparoscopic resection of parapelvic cavernous hemangioma.

**Case presentation:**

A 46-year-old female, who diagnosed as parapelvic cavernous hemangioma accompanying with thrombosis and calcification, was performed laparoscopic resection using DaVinci surgical system under general anesthesia. The patient well recovered without recurrence or spread of the lesion after operation for 3 months as well as hydronephrosis was significantly relieved.

**Conclusion:**

Laparoscopic resection of parapelvic cavernous hemangioma under the help of DaVinci surgical system was feasible and safe.

## Background

Hemangiomas are vascular tumors and more commonly in infants, originating from the residual embryonic angioblasts, containing proliferative vascular endothelial cells, with angiogenic phenomenon [1]. Hemangiomas are more commonly in skin, mucous membrane, liver, kidney, intestine, bone and muscle, among which kidney is the second internal organ following liver [2]. Renal hemangiomas have a characteristic presentation of abdominal mass, pain and hematuria [3]. Cavernous hemangioma is a rare, non-functional, benign adrenal tumor. Due to it usually shows no signs or symptoms and only noted when it developed a large palpable mass or spontaneous rupture causing to hypovolemic shock of patients, it is difficult to distinguish from retroperitoneal lymphoma and paraganglioma.

Traditional surgical treatment should not be used as the first choice of treatment for hemangiomas. Due to the deep location of hemangioma, open surgery may require a lot of free organs around hemangioma, such as vena cava, duodenum, liver, adrenal gland and others, which may increase risk of additional damage. Conventional laparoscopy increases the difficulty of surgery because of the visual field is not three-dimensional (3D) view and the surgical instruments are not flexible with robotic arms. DaVinci surgical system was applied under the help of robots which has been common used in surgery and exhibit great superiority. Robots could improve dexterity and reduce operator fatigue compared to surgeons, as well as with a 3D view which provides an unmatched view of anatomical structures [4]. For example, robotic-assisted hysterectomy could gained more advantages, such as improving dexterity, coordination, and visualization compared with the conventional laparoscopy [5]. At the same time, the application of robotics was boosted in urology as main procedure for prostate cancer [6, 7]. Overall, robotic technology allows the surgeon to perform complex tasks in a minimally invasive fashion [8]. Due to the lack of specific manifestations of renal hemangioma in imaging, the robotic surgical method can be used for diagnostic treatment with small surgical trauma and improve the correct diagnostic rate [9].

In the present study, we proposed a case which successfully resected parapelvic hemangioma under the help of robotic-assisted device. Postoperative examination confirmed that this kind of hemangioma was cavernous hemangioma. The surgical management of parapelvic hemangioma need complex technical requirements, which can increase the difficulty of surgery. The aim of this article was to improve the level of diagnosis and treatment of parapelvic hemangioma assisted by robots.

## Case presentation

**Fig. 1:**
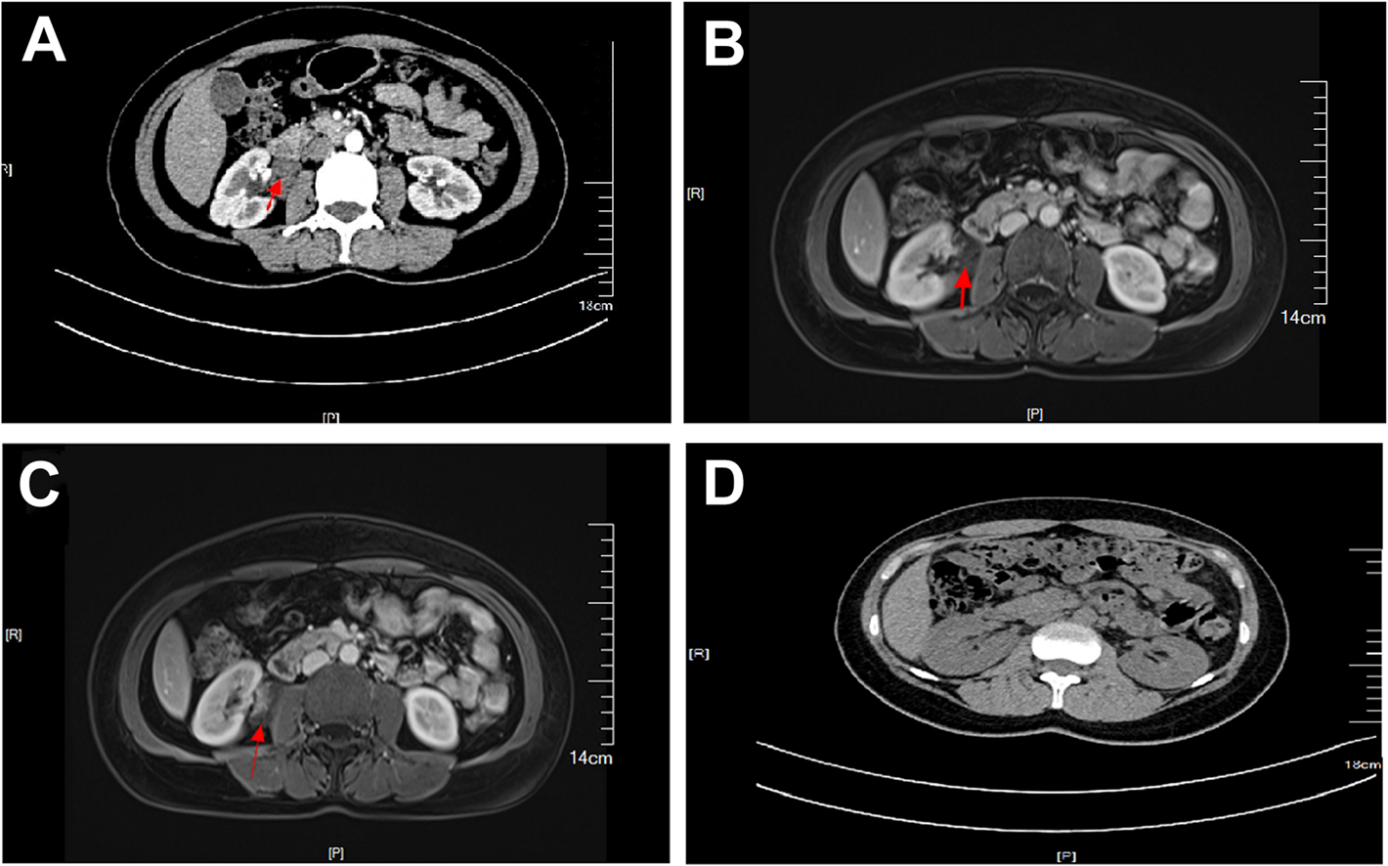
**a** The CT image of cavernous hemangioma; **b** The MRI image of cavernous hemangioma; **c** The enhancement scan image of the cavernous hemangioma; **d** The CT image of the patient after discharging 3 months

A 46-year-old woman of Han nationality, who had a history of appendectomy 2 months ago, was admitted to hospital again on April 1, 2019, due to had a pain on the right side of the waist for more than 20 days. Physical examination showed no abnormities in head, chest, abdomen and limb activity. There was no mass in the right kidney and the patient felt pain when tapping on her kidney. Routine blood test showed that the number of erythrocyte and leukocyte were 3.7 × 10^12^/L and 7.37 × 10^9^/L, respectively, and the level of hemoglobin was 108 g/L. Urine routine test showed that erythrocyte and leukocyte were 44.7/ul and 361.2/ul, respectively. Computerized tomography (CT) revealed an ill-defined soft tissue mass on the junction regions of ureters and right renal pelvis, the lesion were obviously continuously and unevenly enhanced and with calcified margins, and enlarged hydronephrotic right renal pelvis (Fig. 1a). Magnetic resonance imaging (MRI) showed the ill-defined soft tissue mass of a size of 3.1 cm × 2.3 cm × 4.0 cm with an unclear margin (Fig. 1b). Enhancement scan showed significant and continuous uneven mass, limited diffusion weighted imaging (DWI), high signal intensity on apparent diffusion coefficient (ADC) map, the lesion had unclear margin with right renal collecting system, the right renal pelvis and partly ureter moved to forward, and enlarged hydronephrotic right renal pelvis (Fig. 1c). Other examinations, including electrocardiogram (ECG), chest film, biochemistry analysis, coagulation, blood cortisol, vanillylmandelic acid (VMA), renin activity, angiotensin I, angiotensin II, epinephrine and noradrenaline, were all normal. Preliminary diagnosis was therefore right parapelvic mass.

**Fig. 2:**
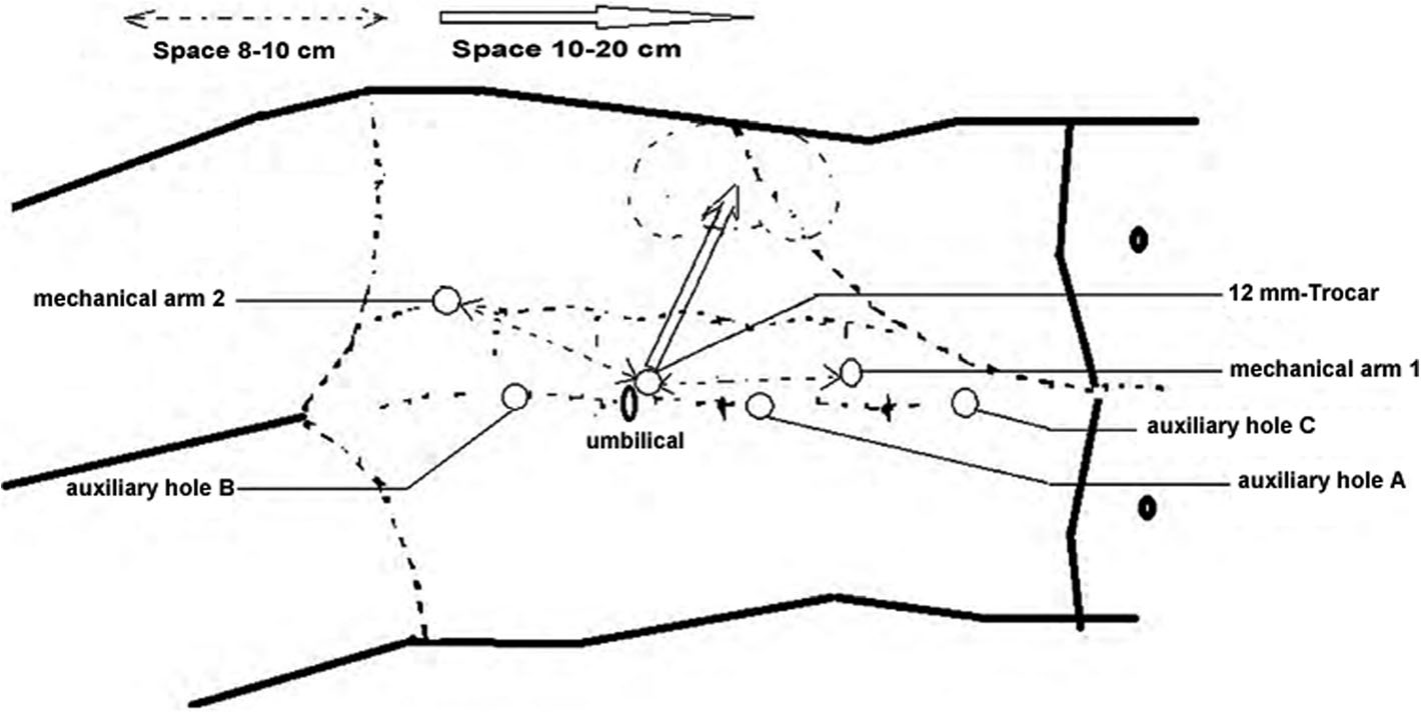
Schematic diagram of Trocar site

**Fig. 3:**
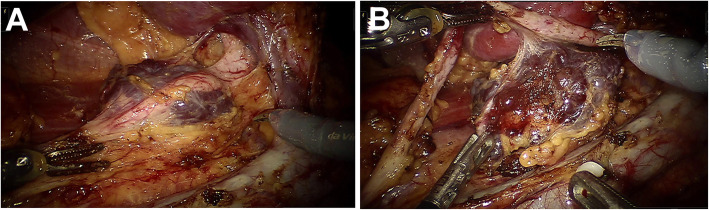
**a** Image of identifying the blood supply; **b** Image of tumor resection


On April 16, 2019, after general anesthesia, the patient was placed in the left lateral position and performed DaVinci robotic-assisted laparoscopic resection of parapelvic lesion via the transabdominal approach. A small incision about 1 cm was made at the umbilical margin of the affected side with a Veress needle to establish pneumoperitoneum at pressure of 12–15 mmHg. Then, a 12 mm-Trocar was inserted into abdominal cavity through the incision (Fig. 2). Two mechanical arms were located at a distance of 8–10 cm to the lens hole to control various instruments: the mechanical arm 1 (8 mm) was 2–3 cm to the twelfth rib; the mechanical arm 2 (8 mm) was on 15 ° angle and 2–3 cm medial to the anterior superior iliac spine. Auxiliary holes A and B were established 5 cm outside the midpoint of the connection between the camera arm and mechanical arm 1 and 2, respectively. Due to the right side lesion, a 5 mm-Trocar was placed under the xiphoid process to lift the liver in auxiliary hole C. The next measures were to loosen the adhesion in the abdominal cavity, dissect the parietal peritoneum along the paracolic sulcus, push the ascending colon to the opposite side, dissect the ligament of the liver and colon, insert the needle holder with the self-locking device into the auxiliary hole C under the xiphoid, and lift the lower edge of the liver. The electric scissors was used to sharply separate the anatomical space between the inferior vena cava and the duodenal descending fusion fascia. After pushing the duodenum to the medial side, perirenal fascia and the tissue on the surface of the renal pelvis were opened, and the upper ureter was separated along renal pelvis. Purple/brown hemangioma-like neoplasm with a diameter of about 4 cm was observed in the upper segment of the right ureter and behind the right renal pelvis, it surrounded the junction of right renal pelvis and ureter, and its blood supply originated from the vessels in the right renal sinus (Fig. 3a). The cavernous hemangioma was completely removed through DaVinci robotic-assisted laparoscopic resection following the steps in the video (see Supplementary Video, Fig. 3b). The surgery is uneventful and the patient was followed up until now without any complications (Fig. 1d).

**Additional file 1: Supplementary Video.** The cavernous hemangioma was completely removed through DaVinci robotic-assisted laparoscopic resection following the steps in the video.

## Discussion

The occurrence of hemangiomas is related to sex hormone levels, such as estrogen or progesterone [10]. Cavernous hemangioma, serving as a special renal hemangioma, is consist of many thin-walled vessels, which is vascular malformation lacking arterial composition [11]. Cavernous hemangiomas near the renal pelvis are rare, which is easily to be misdiagnosed as ectopic adrenal mass, ganglioneurocytoma, and castleman disease [12–14]. In recent 10 years, no more than 10 cases of hemangioma of renal pelvis were reported in China. Renal cavernous hemangioma of the kidney is difficult to diagnose preoperatively [15]. It will be found only when the tumor being large to press the adjacent organ. In the present case, cavernous hemangioma occurred posterior side of the junction between the pelvis and ureter and part of the tumor enveloped the ureter leading to hydronephrosis.

DaVinci surgical system has been applied around the world, it inherits the advantages of minimally invasive laparoscopic surgery, but also makes the surgical field of stereoscopic enlargement. The robot arm can complete 540 degrees of rotation, movement, swing and grip in the narrow anatomical area of the human body. It has the stability, reproducibility and accuracy that human hands cannot reach, and can assist to complete various delicate and complex operations [16]. For example, organs in urinary system are mostly with the deep location and narrow space, which are suitable to use a robotic surgical system to perform minimally invasive surgery [17]. Furthermore, the robot-assist surgery could reduce trauma, shorten the length of hospital stay and benefit to the recovery of patients.

The deep location of lesion and unclear operative field are all difficulties for the renal cavernous hemangiomas surgery. Before, the robot-assisted laparoscopic surgery to resect cavernous hemangiomas near to renal pelvis are rarely reported, this case was firstly reported. Under the magnification lens and the 3D imaging technology of the robot, the renal artery and renal vein at the renal hilum were clearly revealed in clear intraoperative vision. We found that positioning the puncture point and docking the robot system were the keys of the surgery. The distance between the lens cannula point and robot arm cannula point should be at least 8–10 cm, as well as the distance between these cannula points and the surgical area should be at least 10–20 cm, which would provide the maximum area for the surgical instrument and avoid collisions between external robotic arms. In addition, the proper distance between the lens cannula point and the operation area was 10 cm. A distance greater than 10 cm might make the field of view too far for the surgical instruments to reach the operation area. Although the robotic arm is flexible and easy to operate, the surgeon needs to conform that the robotic arms were in the field of vision to ensure the safety of the operation. Under the guidance showed above, the operation was successfully completed for the tumor was completely removed along the tumor envelope. In conclusion, laparoscopic resection of parapelvic cavernous hemangioma under the help of DaVinci surgical system was feasible and safe.

## Data Availability

All data generated or analyzed during this study are included in this published article.

## Abbreviations

CT: Computerized tomography
3D: Three-dimensional
MRI: Magnetic resonance imaging
DWI: Diffusion weighted imaging
ADC: Apparent diffusion coefficient
ECG: Electrocardiogram
VMA: Vanillylmandelic acid
